# Aggregates mineralogical composition dataset to estimate the averaged aggregate hardness parameter to predict the long-term skid resistance of pavements

**DOI:** 10.1016/j.dib.2020.105849

**Published:** 2020-06-11

**Authors:** Malal Kane, Vikki Edmondson

**Affiliations:** a Université Gustave Eiffel, Campus de Nantes, Allée des Ponts et Chaussées, 44340 Bouguenais, France; b Northumbria University, Civil Engineering, Engineering & Environment, Newcastle upon Tyne, NE1 8ST, United Kingdom

**Keywords:** Long-term skid resistance, Asphalt surfacings, Aggregates mineralogical composition, Petrographic nature, Aggregates types, Averaged Aggregate Hardness Parameter, Polishing, Traffic

## Abstract

The raw data here are used to calculate the AHPM (“Averaged Aggregate Hardness Parameter”) parameters of pavement surfaces and to determine their capacity of skid resistance in the long term. They are composed by:

• the type of aggregates and their proportions by volume in each pavement,
• the calculation of the Aggregate Hardness Parameter (AHP) and
• the determined AHP of each of the pavements.

After the calculation of this parameter and with the help of analytical functions that we recall below, the skid Resistance capacity of that asphalt surfacing in the long term will be deduced. This long-term skid resistance value corresponds to that determined in the test with the Wehner Shulz machine.

The reader is invited to read the paper entitled Long-Term Skid Resistance of Asphalt Surfacings and Aggregates’ Mineralogical Composition: Generalisation to Pavements made of Different Aggregate Types referenced WEA203339 [1].

Specifications Table

**Table:** 

Subject	Civil and Structural Engineering
Specific subject area	Pavement engineering. The work aims to find the relationship between the types of coarse aggregates used in asphalt mixes and the long-term skid resistance capacity of the resulting pavements.
Type of data	Table
How data were acquired	Petrographic examination of aggregate samples was carried out under BS EN 932–3: 1997 [Kane et al., 2013].
Data format	Raw
Parameters for data collection	The general characteristics of the aggregate samples including maximum particle size, texture, and shape were examined and recorded. The main rock types were then identified and the relative proportions of the mineral constituents were estimated using a light (optical) microscope. Colour, grain size, and degree of weathering were also recorded.
Description of data collection	To facilitate the quantitative examination, aggregate samples were sieved into separate size fractions and the mass of each size fraction determined. Each size fraction was then examined and the petrological composition was determined by hand separation and weighting [2,3]. The method employed required two representative samples to be tested, with the result taken as the mean of the two measurements.
Data source location	Université Gustave Eiffel Campus de Nantes Allée des Ponts et Chaussées, 44,340 Bouguenais, France
Data accessibility	With the article
Related research article	M. KANE, V. EDMONDSON, Long-Term Skid Resistance of Asphalt Surfacings and Aggregates’ Mineralogical Composition: Generalisation to Pavements made of Different Aggregate Types, WEAR, In Press

Value of the Data

This data is interesting because it allows you to follow the calculation procedure that leads to the parameter AHP.
Anyone involved in asphalt mix design can use this data to predict the long-term skid resistance of his future surface.
These data can be used and supplemented by other petrographic analyses of aggregates not included in this list.

## 1 Data description

The file Raw_Data.xls is an excel file containing:

• The Sample characteristic including the type of aggregate and proportions by volume and the long-term skid resistance measured on these samples. The first letters A and M of the names of the samples gives their natures (A for Asphalt mixes and M for Mosaic) (Table 1),
• The Mineral Composition of the aggregate contained in the samples (Table 2),

## 2 Experimental design, materials, and methods

Petrographic examination of aggregate samples was carried out under BS EN 932–3: 1997 [Kane et al., 2013]. The general characteristics of the aggregate samples including maximum particle size, texture, and shape were examined and recorded. The main rock types were then identified and the relative proportions of the mineral constituents were estimated using a light (optical) microscope. Colour, grain size, and degree of weathering were also recorded. To facilitate the quantitative examination, aggregate samples were sieved into separate size fractions and the mass of each size fraction determined. Each size fraction was then examined and the petrological composition was determined by hand separation and weighting [BS EN 932–3: 1997, Kane et al., 2013]. The method employed required two representative samples to be tested, with the result taken as the mean of the two measurements.

## Declaration of Competing Interest

The authors declare that they have no known competing financial interests or personal relationships that could have appeared to influence the work reported in this paper.
